# The Recent Conventions

**Published:** 1895-10

**Authors:** 


### Editorial.

### THE RECENT CONVENTIONS.

A SUFFICIENT time has elapsed to form an unprejudiced opinion in regard to the work of the several national organizations that met this year at Asbury Park, New Jersey. These meetings would be valueless did they not open up to a more enlarged conception of professional duty and to a broader foundation in professional knowledge.

That this much is accomplished in all large gatherings must be conceded, and this constitutes the best reason for their continuance.

While this is true it by no means follows that this widening of scope has been extensive, or that the horizon, in a scientific direction, has been greatly enlarged ; indeed, were we to express in a few words the outcome of these gatherings, we should say that, beyond the minimum effect, there had been nothing gained by the assembling of delegates from all parts of the country. This remark applies with peculiar force to the meeting of the American Dental Association.

It is well understood that criticism of large bodies is never received kindly, for the excellent reason that whatever may be their short-comings the managers are rarely or never to blame. It is unreasonable to expect a national gathering to rise higher than its source of supply, and the dental profession, in its entirety, is not equal to the formation of a body thoroughly capable of grasping problems with scientific accuracy. Hence there may be something of harshness when it is stated that, in the opinion of the writer, the meeting of the American Dental Association of this year was no better than many that have preceded it; in fact, was not up to the standard of a well-organized local association. This would seem to point to the probability that national associations have had their day, and that the real work of the profession will, in the future, be accomplished by local organizations.

That this is true is manifested by the continual infliction of papers of but little general interest and of doubtful practical application. Those who were present at the recent meeting will recall the time taken up by the reading of three papers on nomenclature when one would have been ample. The subject is one of some interest in an educational sense, but, as has been repeatedly stated in these pages, language cannot be changed by essays, however learned they may be.

So much time was absorbed in the reading of these and others similarly duplicated that many of the sections were practically ignored and the papers read by title. This practice is unjust to the writers and a possible loss to those in attendance. The fault lies in the absence of a properly organized head to this body. The Executive Committee has no opportunity to learn, as far as we are aware, how many papers are to be presented or what will be their character. This is left, principally, to the chairmen of the various sections, who make their reports according to the order of business. The result is a lack of systematic arrangement, which gives a mass of material impossible to present or to discuss. It is probable that those present may not have suffered any loss by title reading, but it is certainly not a dignified way of conducting meetings, nor does it add any to the interest.

The question has been repeatedly asked, why the attendance was not greater? The proximity to four of our large cities, in which some of the most active minds in dentistry reside, seemed to warrant the expectation of a very large meeting, and yet the number, while large, was not much greater than at less favorably situated resorts. This paucity of numbers can only be accounted for upon the basis of a lack of interest. The proceedings, as published from year to year, have not aroused sufficient enthusiasm to induce unusual efforts or sacrifices. This is unfortunate, for, if nothing else be gained, the social intermingling is above price, leading to a greater infusion of energy in the upbuilding of the profession. Nothing so certainly leads to retrogression as isolation. Commingling means friction and friction means growth and increased strength.

Great improvements are necessary in this Association if it is to remain worthy the attention of the scientific thought in dentistry. It will not be much of a gain to adopt the plan as proposed by the Executive Committee that the sections meet daily. These branches of the main body, as at present organized, are of but very little use, except as a channel by which the main body can be fed. If, however, it is meant by this proposition to make sectional meetings for the discussion of papers, then we beg leave to enter a serious protest. This plan was tried at Chicago to the general disgust of all those in attendance, and should never be repeated. What is required is a central power with the moral courage to eliminate matter not wanted. Reduce the number of papers. Insist on synopses being furnished and printed prior to the meeting, and give time for the expression of opinions for or against the ideas advanced. Give preference always to original work, and let such papers be read at the beginning of the meeting and not left to the last when there is scant time either to hear or discuss. If the iron hand of revision could be laid upon the productions in advance of reading, it would help to a clearer understanding of the subject-matter.

It seemed a fateful combination of circumstances that the American Dental Association should be, at this meeting, entirely surrounded by trade. It has been the feeling of many good men that unless this trade interest was eliminated dentistry would never rise to the dignity of a profession, and here we had its influence exhibited to an unbearable degree. The auditorium, where the meeting was held, was unusually large, thus enabling the local committee to give ample space for an exposition of goods of varied character from an inkstand to a dental engine. It was certainly a remarkable exhibit, and the visitors at Asbury Park were not slow in discovering it. The meeting proper was held inside of this *fringe* of trade, which almost completely surrounded and dominated it. The noise of the visiting multitude made attention to the work of the convention a serious labor, and produced much dissatisfaction. It is to be hoped that the Executive Committee will see to it that this unpleasant combination is avoided in the future. We regarded the selection of this hall a mistake from the first, being too large for a comfortable meeting. Educational Hall would have been preferable in every respect, and the trade interests, valuable as they always are, could have been exhibited in an adjoining and very convenient building.

The work of the associations meeting at the same place, such as the National Association of Faculties, National Association of Examiners, School of Dental Technics, and Dental Protective Association, will be reported in regular order.

The "Faculties" made but little advance in the standard of dental education. It was anticipated that the course would be extended to seven months, but, for reasons best known to the majority, this was not adopted, and a compromise on a six months' course was finally decided as the best attainable at the present time.

We have no desire to give undue expression to a conviction that this organization has need of more wisdom than was manifest at the recent annual meeting. An element seems to have entered its counsels which, if not eliminated, will most assuredly end in its disruption. This, should it occur, would be a calamity, for in union only will be found strength.

The Dental Protective Association held an enthusiastic meeting and one that should be productive of far-reaching results. It is doubtful whether the majority of those present were ever before as fully informed of the working of this organization or so generally impressed with the unselfish work of Dr. Crouse, its founder and manager. It was the general impression that if this information were made general the profession would take a deeper interest. It is, unfortunately, true that the majority of dentists have entertained prejudices against the management, regarding it as altogether too personal. This has been exceedingly unfortunate, and has acted as a serious bar to the progress of the Association, and it is hoped that the publishing of the proceedings of this meeting will go far to disabuse the minds of many of the erroneous idea alluded to and lead to a more active co-operation.

The School of Dental Technics had a full meeting, and considerable interest was manifested in its work. This, to our conception, is not sufficiently extended to warrant a separate organization. It might, with very great propriety, be merged into the Association of Faculties, for that body will be forced, in the near future, to take up the consideration of methods of teaching, it having very nearly completed the preliminary work it set itself to accomplish.

The spirit of antagonism to dental colleges, which yearly makes itself felt in all our conventions, was especially obnoxious at the American Dental Association. We cannot believe that the sentiments expressed are shared by the majority, for, if this were true, dental colleges will have had their day. The presiding officer should prohibit the use of the floor upon these occasions for the expression of malignant spite and libellous accusations. It is to be hoped we have seen the last of these humiliating exhibitions.

The criticism made against Asbury Park as a place of meeting applies with equal force to all summer resorts. It is to be regretted that the Association selected Saratoga Springs for the next meeting, for this place is in no degree better, for the members are forced to scatter all over the town, destroying the possibility of social intercourse, the lack of which was the most objectionable feature in the recent meeting. The true locality for these associations is Niagara Falls, and it is to be hoped that the Saratoga convention will settle upon this favored place for the succeeding five years, and thus avoid the yearly migration to unsatisfactory locations.

It was a matter of profound regret that the honored president of the American Dental Association, Dr. J. Y. Crawford, should have been stricken down with a severe illness at the close of the first day. He had the sympathy of the entire meeting, which was manifest by re-electing him president for the ensuing year. His sickness, while severe and prolonged, ended happily in his recovery. The duties of the presiding officer were most satisfactorily performed by the vice-presidents, Dr. S. C. G. Watkins, of New Jersey, and Dr. Thomas Fillebrown, of Massachusetts.

The work of preparing for this meeting was voluntarily assumed by the New Jersey State Dental Society, and, while some things might have been omitted to advantage, nothing but praise is due for the great labor and intelligent efforts expended to make the meeting a success.

It is to be regreted that so many fail to meet at these gatherings. If nothing else be gained, there ensues a co-operation of interests in the active commingling, and this is an immeasurable benefit to the individual and to the society. The influence of every one is needed that dentistry may progress, and no one has a moral right to withhold it. Let, therefore, Saratoga be the Mecca of all thoughts for the coming year, and gather by its sparkling waters, and there renew strength and add to the vitality of the profession to which we all owe allegiance.

